# DNA Methylation in T-Cell Acute Lymphoblastic Leukemia: In Search for Clinical and Biological Meaning

**DOI:** 10.3390/ijms22031388

**Published:** 2021-01-30

**Authors:** Natalia Maćkowska, Monika Drobna-Śledzińska, Michał Witt, Małgorzata Dawidowska

**Affiliations:** Institute of Human Genetics Polish Academy of Sciences, 60-479 Poznań, Poland; natalia.mackowska@igcz.poznan.pl (N.M.); monika.drobna@igcz.poznan.pl (M.D.-Ś.); michal.witt@igcz.poznan.pl (M.W.)

**Keywords:** T-cell acute lymphoblastic leukemia, T-ALL, DNA methylation, epigenetic aberrations, epigenetic prognostic markers, methylation phenotypes in cancer

## Abstract

Distinct DNA methylation signatures, related to different prognosis, have been observed across many cancers, including T-cell acute lymphoblastic leukemia (T-ALL), an aggressive hematological neoplasm. By global methylation analysis, two major phenotypes might be observed in T-ALL: hypermethylation related to better outcome and hypomethylation, which is a candidate marker of poor prognosis. Moreover, DNA methylation holds more than a clinical meaning. It reflects the replicative history of leukemic cells and most likely different mechanisms underlying leukemia development in these T-ALL subtypes. The elucidation of the mechanisms and aberrations specific to (epi-)genomic subtypes might pave the way towards predictive diagnostics and precision medicine in T-ALL. We present the current state of knowledge on the role of DNA methylation in T-ALL. We describe the involvement of DNA methylation in normal hematopoiesis and T-cell development, focusing on epigenetic aberrations contributing to this leukemia. We further review the research investigating distinct methylation phenotypes in T-ALL, related to different outcomes, pointing to the most recent research aimed to unravel the biological mechanisms behind differential methylation. We highlight how technological advancements facilitated broadening the perspective of the investigation into DNA methylation and how this has changed our understanding of the roles of this epigenetic modification in T-ALL.

## 1. Introduction

Aberrations affecting genes involved in the process of DNA methylation and the resulting aberrant methylation signatures have been observed across solid tumors and hematological malignancies. These include aberrations in the genes encoding elements of the epigenetic machinery itself (epigenetic modifiers) and in the genes functionally upstream of the modifiers (epigenetic modulators) encoding the activators and repressors of the epigenetic machinery, which activity changes in response to environmental factors and aging [1]. These lead to altered methylation, and thus altered expression of genes called epigenetic mediators. Considering critical roles of DNA methylation in embryonic, germline and somatic cell development [2], it is postulated that many of the epigenetic mediators are the genes responsible for proper maturation programs; disruption of these programs upon epigenetic changes contributes to cancer development [1].

T-cell acute lymphoblastic leukemia (T-ALL) is an aggressive hematological malignancy characterized by a complex (epi-)genetic molecular background, including chromosomal aberrations, copy number alterations (CNA), DNA sequence mutations as well as aberrant gene expression and methylation profiles [3]. T-ALL arise in the thymus from T-cell precursors (thymocytes). Normal differentiation and maturation of thymocytes is abrogated and taken over by malignant proliferation as a result of the accumulation of genetic and epigenetic lesions.

Over the years numerous genetic abnormalities in T-ALL have been identified, including chromosomal rearrangements involving T-cell receptor (TCR) loci, activating mutations of *NOTCH1*, 9p21.3 deletions associated with impairment of *CDKN2A/2B* cell-cycle regulators and ectopic expression of several transcription factors, acting as oncogenes, including: *TAL1, LYL1, TLX1/3, HOXA9/10, NKX2-1* and *LMO1/2* [3]. In the past decade, the systematic screening of T-ALL genomes by next generation sequencing and high resolution arrays have revealed a wide spectrum of novel genetic aberrations, some of which are considered as potential prognostic markers or therapeutic targets. Yet, thus far none of these genetic features has been widely introduced into T-ALL therapeutic protocols as risk stratification factors. Hence, the field of epigenetics, particularly DNA methylation, has been extensively explored in search for aberrations with clinical significance in T-ALL.

Here we present the current state of knowledge on the role of DNA methylation in T-cell development with the focus on epigenetic aberrations contributing to T-ALL pathogenesis. We further review the research investigating the existence of distinct methylation phenotypes observed in T-ALL patients (hyper- and hypomethylation) related to different clinical outcomes and most likely resulting from different mechanisms underlying T-ALL development in these groups. We highlight how technological advancements facilitated the widening of the investigation into DNA methylation and how this broadening perspective has changed our understanding of the roles of this epigenetic modification in T-ALL.

### DNA Methylation: Basic Aspects and Biological Roles

DNA methylation is based on covalent transfer of methyl groups (-CH_3_) to nucleotide nitrogen bases. Addition of the methyl group to the 5th carbon of cytosine results in the generation of 5-methylcytosine (5mC) [4]. DNA methylation is particularly frequent in the palindromic CpG sites, which is important in maintaining the methylation profile of a daughter DNA strand during cell divisions [5]. Less frequently this modification may also occur in non-CpG context [6,7].

A group of enzymes called DNA methyltransferases (DNMTs) generate and maintain genomic methylation profile, and are frequently referred to as DNA writers [4]. The DNMTs family consist of four proteins with partially different functions. DNMT3A and DNMT3B conduct de novo methylation, establishing DNA methylation pattern at early stages of embryogenesis, while DNMT1 is responsible for maintaining the methylation pattern by copying the methylation marks into the daughter DNA strand under the S phase of mitotic cell divisions. DNMT3L alone does not possess any inherent enzymatic activity, but can activate DNMT3A and DNMT3B by binding to their catalytic domains. Of note, the DNMTs were discovered to introduce the toxic 3-methylcytosine (3mC) lesions to DNA [8]. Although this process is strictly controlled by DNA repair enzymes, the repair mechanisms may fail, increasing mutational rate and potentially contributing to pathogenic transformation.

Over the years, methylation was considered to be an irreversible DNA modification due to the strength of the carbon-carbon bond, but its active erasing has been confirmed in 2010 [9,10]. Active demethylation in mammals is initiated by ten-eleven translocation (TET) dioxygenases, which belong to DNA methylation erasers [11]. In human, three TET paralogs have been identified, namely TET1, TET2, TET3 [12]. TETs oxidize the 5-methyl group of 5mC, converting it to 5-hydroxymethylcytosine (5hmC) [13], in the next step oxidize 5hmc to 5-formylcytosine (5fC) and finally to 5-carboxylcytosine (5caC) [14]. Subsequently, 5caC undergoes glycosylation by thymine DNA glycosylase (TDG) and unmodified cytosine is introduced by base excision repair (BER) pathway [9,15].

Despite the evolutionary ancient origin of cytosine methylation, this mechanism is absent in few eukaryotic species. This phenomenon is related to highly mutagenic nature of 5′ cytosine methylation as 5mC can spontaneously undergo deamination, leading to C → T transitions in the genome [16,17]. For this reason, mammalian genomes have approximately 5-fold less of CpG dinucleotides then it is expected by random genome composition.

The distribution of CpG sites within the mammalian genome enables to define CpG islands (CGIs), CGI shores and CGI shelves. CpG islands are regions with higher frequency of CpG dinucleotides than average in the genome (an observed to expected CpG dinucleotide ratio of ≥0.60). The majority of CGIs are at least 200 bp up to 1kb long and have >50%, usually 60–70% content of C + G [18]. Many mammalian gene promoters are associated with CGIs, which when unmethylated are linked to active transcription. It is the methylation of CpGs in gene promoters that is an epigenetic mechanism of transcriptional silencing [18]. The CGI shores are sequences immediately flanking CGIs, defined by 2 kb up- and downstream from CGIs. Beyond them there are more distant regions (the next 2 kb from CGIs) called CGI shelves. The CpG sites located in the remaining parts of the genome belong to the so called ‘open sea’ regions [19]. The CGI flanking regions have comparatively low CpG dinucleotide density and have been linked to the regulation of gene expression via regulation of transcriptional elongation, determination of alternative promoters [20], regulation of mRNA splicing [21] or affecting the binding of transcription factors with enhancer regions [22]. The methylation of CGI shores have been shown to be associated with high gene expression, particularly in cases of highly methylated shores flanking unmethylated CGIs, stable across tissue types, developmental stages and disease states [23]. Such stably methylated CGI shores have been found in proximity of many housekeeping genes [23]. Of note, the majority of tissue-specific DNA methylation and also most of the cancer-specific DNA methylation (as shown in colon cancer) does not reside in CGIs but in CGI shores and affect development-related and pluripotency-related genes [24]. These findings support the notion that epigenetic alterations mainly contribute to cancer development by affecting cells’ differentiation.

Physiologically, mammalian genomes have a level of CpG methylation oscillating around 70–80% with some tissue- and age-specific differences [25,26]. The 5mC primarily occurs in constitutive-heterochromatin regions and within inactive gene promoters [27]. In contrast, methylated DNA regions which occur within gene bodies are associated with transcriptional activity and probably have roles in the regulation of splicing with the involvement of methyl-CpG-binding proteins [21].

DNA methylation plays important roles in key biological processes, such as the regulation of stability of chromosomal structure, segregation of chromosomes during mitosis, transcriptional repression of retro-elements, monoallelic silencing of imprinted genes and regulation of gene expression in a spatiotemporal-specific manner [28]. Disturbances of the physiological processes of methylation and demethylation are of critical consequences for the development of cancer. Ectopic expression of the genes crucial for cell fate decisions during cell differentiation or aberrant levels of these genes’ expression, contribute to malignant reprogramming of the cells.

In general, as compared to normal cells, cancer epigenomes are characterized by global hypomethylation of CpG poor regions with local hypermethylation of CGIs within promoter regions [29]. It is assumed that in cancer cells, DNA hypomethylation, e.g., in retro-elements and centromeres results in chromosome instability. Local hypomethylation of promoters and enhancers causes increased expression of oncogenes, while local hypermethylation of such gene regulatory elements, reduce the level of expression of tumor suppressor genes [30]. Precise mechanisms of how the global DNA methylation profile influence gene expression and translate into pathogenic phenotypes, remains to be fully elucidate in many diseases, including T-ALL.

## 2. DNA Methylation in Normal Hematopoiesis and T-Cell Development

Hematopoiesis is a precisely controlled, dynamic process, which leads to generation and maintenance of morphotic blood elements. Over the years, the concept of hematopoiesis hierarchical model, in which the pluripotent and self-renewing hematopoietic stem cells (HSCs) exist on the top of the hematopoietic hierarchy, has functioned as a dogma. Recent advances in single cell technologies has changed the perception of this developmental scheme, pointing to the heterogeneity within HSCs and progenitor populations. The decision about HSCs’ and progenitor cells’ fate is made in response to the sum of environmental stimuli, which is postulated to be largely mediated by the process of DNA methylation [31].

During early stages of hematopoiesis, pluripotency-related genes remain transcriptionally active, while genes associated with specific cell lineages and their functions are epigenetically silenced. Further on, self-renewal and multipotency-related genes become silenced by methylation and lineage-specific genes become transcriptionally active. These changes are triggered by the methylation and demethylation of gene promoters, yet large part of methylation events in human genome occurs also in the CpG poor regions, distant to transcription start sites (TSS) especially in regulatory elements, like super-enhancers [32].

The analysis of murine hematopoietic progenitor cells showed the dynamic methylation pattern with characteristic marks for myeloid and lymphoid lineage. An example of the epigenetic regulation during T-cell development is progressive demethylation of CpG sites in *Lck* gene, which cause its transcriptional activation [32]. This gene encodes a T-cell specific tyrosine kinase, critical for T-cell development and activation via its role in T-cell receptor (TCR) and pre-TCR signaling [33]. Studying methylation profile in early stages of murine hematopoiesis in a single cell resolution, provided data on differentially methylated regions between myeloid and lymphoid progenitor cells associated with the binding sites for lineage-associated transcription factors (TFs), e.g., *Gata1*, *Gata2*, *Lmo2*, *Runx1* or *Pbx1* [34,35]. Moreover, binding motifs of key hematopoietic TFs are found enriched in transcriptionally accessible chromatin regions in subpopulations of hematopoietic stem progenitor cells (HSPCs) [34,36]. These findings support the notion that DNA methylation is an epigenetic regulatory mechanism of lineage-specific differentiation associated with the activity of specific TFs during hematopoiesis. Integrated analysis of gene expression and DNA methylation profile from single cell sequencing allowed to link the specific epigenetic pattern to defined types of cells during hematopoiesis, not only the mature blood cells but also the subpopulations of HSPC. This approach facilitated *in silico* modelling of hematopoiesis with a range of mature morphotic blood elements and their immature transitional stages [34,35]. It also showed that leukopoiesis in general is marked by increased methylation, which might be the way of suppression of myeloid differentiation program in lymphoid precursors [35].

### 2.1. DNA Methylation Writers Involved in T-Cell Development and Malignant Transformation

DNMT3A has been shown to be involved in T-cell differentiation [37]. Conditional *Dnmt3a* knock-out mouse model has been used to study its role in hematopoiesis. The loss of Dnmt3a function results in general methylation abnormalities with both, hypomethylation and hypermethylation in various loci [38]. Further analysis of differentially methylated genes (DMGs) and differences in gene expression pointed to self-renewal and leukemogenesis-related genes to be affected by these epigenetic aberrations. Importantly, none of mice in this study did develop hematological malignancy, which suggested that mutation in *Dnmt3a* is not a potent leukemogenic driver [38]. It was also reported that DNMT3A indirectly regulate human Th1 cell development, by methylation and thus silencing of *STAT4* gene, encoding a TF critical in T-cell regulatory pathways [39]. Somatic mutations within *DNMT3A* have been identified in up to 17% of T-ALL cases in adults and have been associated with the immature subtypes and with the older age of patients with hematological malignancies. *DNMT3A* loss of function mutations have been correlated with poor prognosis, suggesting that the protein may act as a tumor suppressor [37,40,41,42]. In contrast, in pediatric T-ALL, *DNMT3A* mutations are very infrequent [43,44], which exemplifies age-related characteristics of the (epi-)genetic landscape of T-ALL.

The roles of DNMT1 and DNMT3B in T-ALL biology are less understood. High levels of *DNMT3B* have been associated with poor prognosis in acute myeloid leukemia and few B-cell lymphomas [45,46]. The DNMT1 activity has been associated with self-renewal capability of HSCs by restraining differentiation potential of these cells during hematopoiesis [47,48]. In T-cell lineage, DNMT1 activity was reported at different stages of differentiation into regulatory T-cells [49]. The only study thus far, which has demonstrated the involvement of DNMT1 and DNMT3B in T-ALL biology was published in 2017 by Poole et al. [50]. The RT-qPCR and western blot analysis in MYC-driven T-ALL cells (from transgenic mice with ectopic expression of *c-MYC* in hematopoietic lineages) indicated that *DNMT1* and *DNM3B* expression levels were elevated as compared to normal spleen cells from wild type control mice. Chromatin immunoprecipitation assay showed high relative enrichment for c-MYC protein at the *DNMT1* and *DNMT3B* regulatory regions in mouse T-ALL cells, which suggested direct transcriptional regulation. Reduction of the endogenous expression of c-MYC in human T-ALL cell lines (Jurkat, MOLT-4, P-12, CCRF-CEM) resulted in DNMT3B downregulation. Global methylation analysis by reduced representation bisulfite sequencing (RRBS) in murine MYC-driven T-ALL cells with stable knock-down of DNMT3B showed significant global methylation differences as compared to control cells. The authors proposed the model in which overexpressed c-MYC results in the genome-wide DNA hypermethylation via overexpression of DNMT1 and DNMT3B [50].

The dynamic nature of DNA methylation, the existence of characteristic epigenome in malignant cells and frequent mutations of *DMNTs* in various cancers, make the DNA methylation writers potential druggable targets. DNA methylation inhibitors (iDNMTs) approved by the US Food and Drug Administration (FDA) include 5-azacitidine (vidaza, AZA) and 5-aza-2′-deoxycytidine (decitabine, DAC) [51]. These cytosine analogs inhibit DNMT in proliferating cells, prevent the aberrant methylation to be conserved during DNA replication and thus restore the expression of aberrantly silenced tumor suppressor genes [52]. The antitumor properties of iDNMTs result also from their ability to induce double-strand breaks in DNA, leading to cell cycle arrest [53] and from the induction of global hypomethylation, leading to the reactivation of retroviral elements stimulating immune signaling contributing to anticancer immune response [54]. AZA and DAC, approved for the treatment of myelodysplastic syndromes and acute myeloid leukemia hold promise for treatment options in T-ALL [55].

### 2.2. DNA Methylation Erasers Involved in T-Cell Development and Malignant Transformation

TET1 protein has been reported to have dual roles in hematological malignances depending on the cellular lineage. In B-cell malignances it is postulated to act as a tumor suppressor [56], while in T-ALL it has been shown to carry growth-promoting potential when upregulated [57]. This gene is highly expressed in the majority of T-ALL patients and in many T-ALL cell lines. The higher expression of *TET1* is more frequently observed in adolescent and young adult patients [57]. In pediatric T-ALL patients, higher *TET1* expression was found in intermediate and in high risk groups than in the standard risk group [57]. The oncogenic role of TET1 protein in T-ALL was confirmed by Bamezai et al. in 2020 [57]. The group used shRNA to deplete TET1 in human T-ALL cell lines and in patient derived T-ALL cells transplanted to immunocompromised mice to investigate TET1 function in T-ALL development. Both models have shown that knockdown of *TET1* resulted in the reduction of leukemic growth. Interestingly, TET1 was not critical neither for normal early T-cell development in Tet1-knockout mice, nor for expansion of normal human T-cells in vitro. The authors postulated that TET1 is involved in T-ALL development by maintaining 5hmC marks. They performed hydroxymethylated DNA immunoprecipitation followed by deep sequencing (hMe-DIP)-seq in TET1 depleted JURKAT cells and revealed that downregulation of TET1 resulted in 5hmC decrease at genes associated with cell cycle, DNA repair and oncogenic pathways. What is more, depletion of TET1 was demonstrated to increase DNA damage, as shown by comet assays in cell lines and primary patient T-ALL cells [57]. In the same study the authors demonstrated that *TET1* gets upregulated in response to exposure of T-ALL cell lines to radiation inducing DNA damage. Hence *TET1* was postulated to protect T-ALL cells from DNA damage and thus promote leukemic growth.

Another study has shown that TET1 and TET2 enzymes have distinct functions in T-ALL development; *TET1* acts as an oncogene in T-ALL, while *TET2* is tumor suppressing [58]. Both have MYC binding sites and are regulated by this key oncogene. Aberrantly expressed MYC, acting as a transcriptional activator (in case of *TET1*) and a repressor (in case of *TET2*), deregulates TETs in T-ALL, causing global methylation and hydroxymethylation abnormalities. In all analyzed T-ALL primary samples and cell lines, *TET1* was overexpressed, while *TET2* was downregulated [58]. After MYC inactivation in T-ALL mouse model, the opposite pattern was observed. Restoration of normal *TET1* and *TET2* levels by MYC knockdown in vivo, generated the changes in 5mC and 5hmC profile, and functionally inhibited proliferation of T-ALL cells [58].

Somatic mutations affecting *TET* genes have been reported in hematological malignances [59,60,61,62,63]. Yet these aberrations exerted mild impact on TET proteins expression and global profile of methylation [64,65,66]. Other mechanisms of deregulation or inactivation of TET proteins have also been reported in hematological malignances. These include mutations and epigenetic abnormalities affecting functionally upstream genes (epigenetic modulators). *TET1* has been reported to be regulated via poly (ADP-ribose) polymerase 1 (PARP1) [57,64]. The members of PARP gene family have been shown overexpressed in T-ALL patients, so it is possible that high PARP expression upregulates TET1 via introduction of H3K4me3 marks (euchromatic epigenetic marks) in *TET1* promoter. The available PARP inhibitor, Olaparib, has been demonstrated to reduce *TET1* expression level, to induce loss of 5hmC marks, and to antagonize the growth of T-ALL cells [57]. Hence it proves to be a promising therapeutic agent in this leukemia.

## 3. Investigating Methylation Phenotypes in T-ALL Patients: In Search for Clinical Meaning

Initially, it was the hypermethylation of promoters of tumor suppressor genes that was considered as crucial epigenetic factor in cancer development [67]. Yet, this general assumption, investigated in different cancer types, has been confronted with the findings that methylation profile of tumor suppressor promoters is not uniform and can be differential even within one type of cancer [68]. Studies of several cancer types have shown that certain groups of patients differ by the methylation profile of CGIs within promoter regions, defined as CpG island methylator phenotype (CIMP) [69,70,71]. CIMP+ (hypermethylation phenotype with higher number of methylated loci) and CIMP− (hypomethylation phenotype with lower number of methylated loci) have been observed in glioblastoma, colorectal, breast, lung, and gastric cancers. These findings and a few studies demonstrating the involvement of DNA methylation in hematological malignancies [69,70,71], inspired the research into methylation profiles and their biological and clinical significance in T-ALL patients.

The first study on methylation pattern in T-ALL patients was published in 2005 by Roman-Gomez et al. [72] (Table 1). Although this paper was later retracted due to image manipulation [73] we included it in this review, to present a complete picture of how the perception of methylation has changed in T-ALL. The authors used relatively simple methodology—methylation specific PCR (MS-PCR) to analyze methylation status of 27 promoter regions. These included genes related to cancer (cell immortalization and transformation) and genes previously reported to be methylated in ALL (*CDH1*, *p73*, *p16*, *p15*, *p57*, *NES-1*, *DKK-3*, *CDH13*, *p14*, *TMS1*, *APAF-1*, *DAPK*, *PARKIN*, *LATS-1* and *PTEN*). DNA methylation of selected promoters in T-ALL samples was compared with normal bone marrow and peripheral blood samples as controls. T-ALL patients were classified to CIMP− (samples with up to 2 genes with methylated promoters) or CIMP+ group (with more than 2 genes with methylated promoters). The authors reported that promoter methylation status could impact clinical outcome in T-ALL. CIMP+ patients had higher relapse rate and mortality rate compared to CIMP− [72]. The same group confirmed these findings in a research published a year later, presumably performed in a partially overlapping group of patients [74] (Table 1). Again, the results pointed to a better prognosis of patients with hypomethylation.

The presence of CIMP subgroups in T-ALL was later confirmed by our group (Kraszewska et al. 2011) in pediatric T-ALL cases, with normal bone marrow samples and thymic cells pooled from healthy donors as controls [80]. We also used MS-PCR and investigated a set of 20 genes, partly overlapping with the previous study: *SYK*, *CDH1*, *ADAMTS5*, *NES1*, *FBXW7*, *p16/CDKN2A*, *p73*, *ASPP1*, *NFκB*, *DIABLO*, *sFRP1*, *WIF1*, *CDKN1B/p27KIP1*, *CDKN1c/p57KIP2*, *RB1*, *LATS1*, *BCL11B*, *PTPN2*, *PHF6* and *C/EBPA*. Interestingly, the methylation status of the overlapping genes was not fully concordant between both studies, despite the same methodology. This could be due to sample-specific differences and different CpG spots cover by MS-PCR primer sequences. We found no differences between CIMP groups in terms of clinical characteristics (age and white blood cell counts at diagnosis, initial treatment response, incidence of relapse) nor the distribution of mutations affecting *NOTCH1* and *FBXW7* genes. The main finding of this study was the demonstration, for the first time, that CpG methylation of the studied genes differed between T-ALL cells and normal thymocytes. We concluded, that the observed methylation pattern was specific to leukemia and not to the development of thymocytes. Yet, MS-PCR had an important limitation: it could only serve as a qualitative method for preliminary methylation assessment and not for the quantitative analysis of DMG [80].

The use of microarrays, which encompass a wide range of CpG sites and enable quantitative analysis, was a breakthrough in the understanding of the methylation profiles and their clinical meaning in cancer. The first work which reported the genome-wide promoter methylation status in T-ALL was that by Borssen et al. in 2013 [75] (Table 1). The authors used 27K methylation array, containing 27,578 CpG sites corresponding to almost 14,500 promoter regions. Based on 1347 CpGs most variably methylated among samples, the patients were classified in unsupervised analysis into CIMP+ and CIMP− groups. The majority of differentially methylated sites were located within CGIs. Of note, the DMGs were enriched for targets of polycomb repressive complex (PRC) and for transcription factors, which indicated that CIMP groups mostly differed in their methylation levels of the genes involved in the regulation of developmental and differentiation processes [75]. Interestingly, and in contrast to the previous works [72,74], the authors found significantly better outcome in the CIMP+ group than in CIMP− patients. Thus, technological advancements completely altered the perception of the clinical meaning of DNA methylation in this malignancy.

Different outcomes of T-ALL patients related to CIMP phenotypes were further confirmed by this group in 2016 in an independent cohort of pediatric T-ALL cases [76] (Table 1). A broader spectrum of CpG loci was analyzed using 450K methylation array (485,577 CpG sites). Survival analysis of CIMP classified patients showed significant differences, again with a better prognosis for the hypermethylation phenotype. Yet, the main finding of this study was that CIMP classification added prognostic value to a risk stratification based on minimal residual disease (MRD), being at that moment and still the most reliable prognostic factor in T-ALL [76]. CIMP phenotype enabled more precise classification of patients from high-risk group (defined by MRD > 0.1% by flow cytometry at day 29 of treatment) into: CIMP− patients, who presented with extremely unfavorable outcome and CIMP+ patients with a better prognosis (Table 1). Interestingly, the CIMP phenotype did not influence the outcome of patients with MRD < 0.1%; all patients in this group survived without relapse, regardless of the CIMP classification.

**Figure 1:**
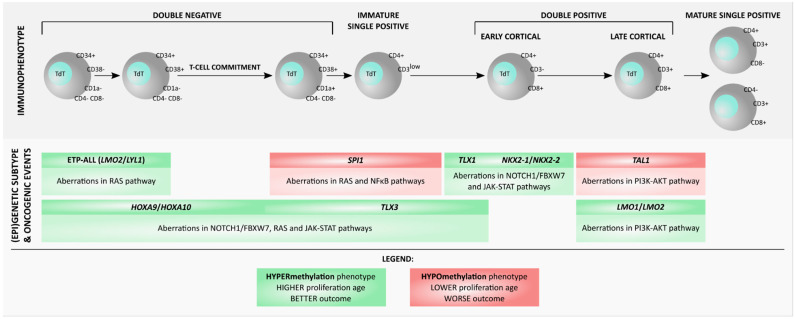
(Epi-)genetic subtypes of T-ALL, based on the findings from reviewed research [75,76,77,78,81,82,83].

The clinical meaning of methylation phenotypes observed in pediatric T-ALL has recently been confirmed in, thus far, the only study in adult T-ALL patients [78] (Table 1, Figure 1). Genome-wide promoter methylation was investigated by methylation-dependent immunoprecipitation (MeDIP) in the discovery cohort, and then by MS-MLPA assay, designed based on the findings from the Me-DIP, applied in a validation cohort. Using the MS-MLPA approach, Inter/High methylation group was identified (corresponding to hypermethylated phenotype) related to better outcome, and Low methylation group (corresponding to hypomethylation phenotype) related to worse outcome (Table 1) [78].

Altogether these studies provided evidence for the prognostic potential of differential DNA methylation in T-ALL and outlined its clinical significance. Interestingly, it was shown in both pediatric [76] and in adult T-ALL [78] that methylation profile was not related to initial response to treatment but it was associated with the increased incidence of relapse in the hypomethylated group. It was shown that patients with hypomethylation phenotype were mostly younger and had higher counts of white blood cells at diagnosis [76,78]. These observations raised the questions on the biological mechanisms underlying differential DNA methylation and its relation to different prognosis in T-ALL. Although a few relevant findings have already been made (e.g., the enrichment of DMGs in PRC target genes) [76], it remained unclear if the hypomethylation was directly involved in the aggressiveness of the disease (e.g., by facilitating leukemia initiation and progression) or was it just a characteristics of the more aggressive T-ALL subtype?

## 4. Investigating Mechanisms behind Methylation Phenotypes: In Search for Biological Meaning

Further technological advancements and the integration of the methylomic data with other ‘omics’ data provided more insights into biological mechanisms related to methylation in T-ALL. The group which previously showed prognostic significance of CIMP phenotypes in T-ALL [75,76] further integrated data from 450K arrays, with targeted exome sequencing and RNA sequencing [83]. They identified associations of methylation phenotypes with previously reported T-ALL driver oncogenes [77,83] (Figure 1). The CIMP− group was enriched in T-ALL cases with increased *TAL1* expression, which was inversely correlated with the methylation of *TAL1* promoter, indicating hypomethylation of *TAL1* promoter as the mechanism of its overexpression. Additionally, *STIL-TAL1* fusions were observed at higher frequency in CIMP− group as compared to CIMP+ cases. This could be explained by the observed hypomethylation of fusion breakpoints, which probably resulted in decreased stability of these regions, making them prone to rearrangements. In CIMP+ group, the higher expression of *HOXA9/10*, *TLX1/2/3* and *NKX2-1* genes were observed, but the mechanisms leading to the upregulation of these genes remained unclear [83]. No genetic lesions nor aberrant promoter methylation have been found to explain the upregulation of these genes. Analysis of DMGs between CIMP subgroups revealed higher expression of G-protein signaling pathway genes in CIMP+ patients and overexpression of genes involved in mTORC2 pathway in CIMP− patients [83]. Additionally, a set of genes of unknown function in T-ALL was identified, *BEX1* and *BEX2* upregulated in CIMP− subgroup; *PLXND1* and *PLCB4* upregulated in CIMP+ subgroup. Yet, the potential roles of these genes in T-ALL have not been examined functionally.

Similar observations on the differential distribution of genetic aberrations between CIMP+ and CIMP− groups were also reported in adult T-ALL patients [78] (Table 1). The CIMP− phenotype was associated with higher incidence of *STIL-TAL1* rearrangements and alterations of *PTEN*, encoding a negative regulator of PI3K-AKT pathway, and lower incidence of *NOTCH1/FBXW7* mutations [84,85].

Most recently two papers were published investigating truly global methylation pattern of pediatric T-ALL using EPIC methylation arrays [77,79] (Table 1). These contain almost twice the number of probes compared to 450K arrays, and cover above 850,000 methylation sites located in: CGIs, non-CpG sites, differentially methylated sites between tumor versus normal cells (identified in various cancer types), enhancer regions, open chromatin regions, transcription factor binding sites and miRNA promoter regions [77].

In the research by Kimura et al., T-ALL samples were classified in unsupervised analysis into: intermediate methylated Cluster 1 and Cluster 2, hypermethylated Cluster 3, and hypomethylated Cluster 4 [77] (Table 1). The EPIC data were integrated with RNA-seq, targeted DNA-seq data and immunophenotype of leukemic cells to investigate the genetic profile of the observed methylation subgroups (Figure 1) [77]. Cluster 1, represented mainly by the more mature late cortical T-ALL immunophenotype, was characterized by *TAL1* gene and PI3K-AKT pathway abnormalities and slightly worse outcome than Clusters 2 and 3. Most cases from Cluster 2 had *TLX* overexpression or early T-cell precursor (ETP) phenotype with mutations in genes related with RAS and JAK-STAT pathways [77]. Interestingly, gene of DEP-domain containing mTOR-interacting protein (DEPTOR) was highly expressed in this group. As shown in human T-ALL cell lines, the expression of *DEPTOR* is activated by NOTCH1 and overexpression of DEPTOR protein increases proliferation of T-ALL cells [86]. The hypermethylated Cluster 3 was characterized by *PICALM-MLLT10* fusions and *DNM2* mutations, related to IL7R/JAK/STAT pathway activation. Cluster 4, grouping patients with global hypomethylation and the most dismal outcome, was enriched in *SPI1* fusions, identified previously by this group (Seki et al. 2017) as a novel driver oncogene in high-risk T-ALL patients [87]. The high *SPI1* expression was correlated with hypomethylation of SPI1 promoter region. The commonly upregulated pathways in Cluster 4, were RAS, NF-κB and cell growth-related genes. Additionally, the authors compared their methylation phenotype classification with the CIMP classification. Clusters 1 and 4 corresponded to CIMP− phenotype and were characterized by worse prognosis, whereas Clusters 2 and 3 corresponded to CIMP+ phenotype and had better outcome [77]. Moreover, global methylation profile of T-ALL samples was compared with that of normal thymocytes. Surprisingly the methylation pattern of hypomethylated Cluster 4 (representing the most aggressive disease) was close to normal thymocytes, while the remaining three clusters were globally more methylated than normal controls [77]. Similar findings were previously reported by Borssen et al. (2016); methylation profile of CIMP− cases was close to normal CD3+ and CD34+ thymocytes, and was interpreted as the indication of a shorter proliferation history of CIMP− as compared to CIMP+ cases [76]. Altogether, these results indicated that aberrant methylation is likely not a driving force of T-ALL onset and progression (the methylation of the most aggressive T-ALL cases is similar to normal controls) but is rather related to the proliferative history of the cells.

This hypothesis was investigated in a recent work by Roels et al. (2020). Based on the EPIC array data a novel classifier was proposed, termed CpG island and Open Sea Methylation (COSMe) [79] (Table 1). Two COSMe subgroups were identified largely based on the methylation of CGIs (referred to as Cluster A regions) which mainly correspond to the promoters of PRC2 target genes. These regions were hypomethylated in COSMe-I and hypermethylated in COSMe-II and strongly overlapped with the regions included in the previous CIMP classification [75,76]. Interestingly, in COSMe-I, these regions were enriched in H3K27me3, a marker of silent chromatin. By integrating the results of DNA methylation with ChIP-seq (chromatin immunoprecipitation and next generation sequencing) and RNA-seq data, it was demonstrated that genes under transcriptional control of CGIs in Cluster A, have no or low expression in T-ALL cases, independently of their methylation phenotype. This indicates that the expression of these genes is silenced either by CGIs hypermethylation, in COSMe-II group, or by H3K27me3 (PRC2-mediated repression) in COSMe-I group [79].

The COSMe classifier also included cluster B and C regions mostly located in gene bodies and intergenic regions. Methylation of cluster B regions could discriminate between immature and more mature T-ALL cases. While methylation pattern of cluster C (corresponding mostly to Open Sea regions) showed the highest heterogeneity between T-ALL cases. The transcripts associated with cluster C showed lower expression in normal T-cells and higher expression in T-ALL samples, and included genes related to T-ALL biology and normal T-cell differentiation [79].

In line with other reports, the hypermethylated phenotype was related to better outcome, while hypomethylation was related to worse prognosis; this analysis was based on the previous criteria for CIMP classification (Figure 1, Table 1) [68,85]. The distribution of aberrations affecting driver oncogenes was also in agreement with previous works: COSMe-I group was characterized by higher frequency of *TAL1* rearrangements, 6q deletions and *PTEN* aberrations, whereas COSMe-II group was enriched in cases with 5q deletions and aberrant activation of *TLX1/3*, *NKX2.1* or *HOXA9* genes (Figure 1) [77,79,83].

Methylation status of Cluster A regions was also investigated to define the proliferative history of leukemic cells [79]. COSMe-I T-ALL cases showed shorter proliferation history and lower mitotic age as compared to COSMe-II cases. This was in line with previous observations [83] on shorter replicative history of CIMP− cases (largely corresponding to COSMe-I) as evidenced by younger epigenetic and mitotic age, and longer telomere length than in CIMP+ cases (corresponding to COSMe-II) [83]. What is more, an increase in the epigenetic age from diagnosis to relapse was observed. It was more profound in COSMe-I cases, indicating higher proliferation rate (contributing to higher aggressiveness) than in COSMe-II [79]. Finally, by the investigation of DNA methylation in two mouse models, with long and short latency of T-ALL, it was demonstrated that the hypermethylation pattern observed in COSMe-II T-ALL is established at the preleukemic state and is associated with the self-renewing capacity of the preleukemic cells [79].

Altogether these findings indicate that in COSMe-I/CIMP− cases, the leukemic onset is faster and the younger mitotic age is reflected by lower methylation. Hence, hypomethylation itself is a result of a shorter proliferative history of leukemic cells, but at the same time it might be considered as a marker of higher aggressiveness of leukemic cells (less time and cell divisions are needed for the overt leukemia to develop). This is in line with higher incidence of inferior genetic features and worse prognosis in the hypomethylated phenotype. In COSMe-II/CIMP+ cases, the disease latency is longer (less aggressive phenotype), which is reflected by higher methylation acquired during the aging of preleukemic cells [77,79,83].

## 5. Summary and Future Perspectives

The research in the recent years have demonstrated that differential DNA methylation is related to prognosis in T-ALL cases (better in hypermethylated and worse in hypomethylated phenotypes). Yet, it has been shown that CpG regions which mainly build the COSMe and CIMP classifiers, correspond to the genes which are not particularly transcriptionally active in T-ALL. Hence, methylation is currently not perceived as the reason for but as the consequence of different dynamics of leukemia onset reflecting different aggressiveness of leukemic cells in these subgroups.

Indeed, methylation phenotypes have been related to genetic aberrations of a putative prognostic value in T-ALL, which might help to define (epi-)genetic subtypes of this leukemia (Figure 1). CIMP−/COSMe-I subgroup is related to higher incidence of *TAL1* [79] and *SPI1* rearrangements and overexpression [79], and to higher frequency of *PTEN* mutations and deletions [79]. Yet, these are just a few selected genes and their associations with methylation phenotypes do not fully elucidate the biological and clinical meaning of DNA methylation in T-ALL. Prognostic value of TAL1 is still unclear; *SPI1* fusions (associated with dismal outcome) are characterized by low frequency in T-ALL and may be specific only for Japanese population [77,87], while the prognostic value of *PTEN* is likely therapy-dependent [88]. Thus, further investigation for genes, pathways and processes deregulated in these subtypes of T-ALL is needed.

The future perspective and an emerging challenge is to integrate the different layers of T-ALL biology, interrogated by epigenomic, genomic, transcriptomic, and proteomic approaches, to unravel the net of biological dependencies behind differential methylation and different prognosis. This is to fully understand the biological meaning of methylation in T-ALL but also to discover genomic and epigenomic aberrations, related to or potentially contributing to the methylation phenotypes observed in T-ALL. Some of the pathways affected by these aberrations might have relevance as therapeutic targets in selected groups of T-ALL patients, potentially identifiable as different (epi-)genomic subtypes, which might pave the way towards predictive diagnostics and precision medicine in T-ALL.

## Figures and Tables

**Table 1:** Overview of studies reporting prognostic potential of differential DNA methylation in T-ALL patients.

Ref.	T-ALL Patients (*n*)	Treatment Protocol	Classifier	Methylation Subgroup	Methylation Phenotype	Outcome	Survival and Relapse Rates #	Classifier Based on Genomic Regions	Method
Roman-Gomez et al. 2005 [72,73]	*n* = 50 (19 pediatric and 31 adults)	PETHEMA ALL-89 PETHEMA ALL-93	CIMP	CIMP−	hypo	better	DFS_12yr_ = 100% OS_13yr_ = 91%	27 selected gene promoters with potential or documented impact on **ALL development**	MS-PCR
				CIMP+	hyper	worse	DFS_12yr_ = 20% OS_13yr_ = 17%		
Roman-Gomez et al. 2006 [74]	*n* = 307 ALL (T- and B-)	PETHEMA ALL-89 PETHEMA ALL-93	CIMP	CIMP−	hypo	better	DFS_12yr_ = 68% OS_12yr_ = 63%	39 selected gene promoters with potential or documented impact on **ALL development**	MS-PCR
				CIMP+	hyper	worse	DFS_12yr_ = 32% OS_12yr_ = 32%		
Borssen et al. 2013 [75]	*n* = 43 (pediatric)	NOPHO ALL 1992 NOPHO ALL 2000	CIMP	CIMP−	hypo	better	EFS_5yr_ = 86% OS_5yr_ = 86%	1038 gene promoters mainly corresponding to **PRC2 target genes**	Infinium^®^ HumanMeth27K BeadArray (Illumina Inc., San Diego, CA, USA)
				CIMP+	hyper	worse	EFS_5yr_ = 36% OS_5yr_ = 45%		
Borssen et al. 2016 [76]	*n* = 113 (pediatric)	NOPHO ALL 2008	CIMP	CIMP + _MRD < 0.01%	hyper	better	CIR_3yr_ = 0% OS_3yr_ = 100%	1038 gene promoters mainly corresponding to **PRC2 target genes**	Infinium^®^ HumanMethylation450 BeadChip (Illumina Inc., San Diego, CA, USA)
				CIMP − _MRD < 0.01%	hypo	better	CIR_3yr_ = 0% OS_3yr_ = 100%		
				CIMP + _MRD ≥ 0.01%	hyper	bette	CIR_3yr_ = 12% OS_3yr_ = 83%		
				CIMP − _MRD ≥ 0.01%	hypo	worse	CIR_3yr_ = 50% OS_3yr_ = 45%		
Kimura et al. 2020 [77] ##	*n* = 79 (pediatric)	n.a.	methylation clusters	Cluster 1	intermediate	worse	DFS_5yr_ ~ 68% OS_5yr_ = 70%	939 most differentially methylated sites within clusters	Infinium^®^ MethylationEPIC BeadChip (Illumina Inc., San Diego, CA, USA)
				Cluster 2		better	DFS_5yr_ ~ 95% OS_5yr_ = 88%		
				Cluster 3	hyper	the best	DFS_5yr_ = 100% OS_5yr_ = 100%		
				Cluster 4	hypo	dismal	DFS_5yr_ = 0% OS_5yr_ = 0%		
Touzart et al. 2020 [78]	*n* = 24 (adult) * n = 168 (adult) **	GRAALL-2003 & GRAALL-2005	nine-promoter classifier **	Inter/High **	hyper	better	CIR_5yr_ = 27% OS_5yr_ = 68%	** 300 differentially methylated gene promoters	* Me-DIP and custom human promoter arrays (Agilent, Santa Clara, CA, USA)
				Low **	hypo	worse	CIR_5yr_ = 45% OS_5yr_ = 50%	** 9 differentially methylated gene promoters selected from Me-DIP results	** MS-MLPA with custom probes
Roels et al. 2020 [79]	*n* = 109 (pediatric)	ALL IC-BFM 2002/2009	COSMe & CIMP	CIMP−	hypo	worse	CIR_3yr_ = 31%	Region A of EPIC array: mainly promoters of **PRC2 target genes**	Infinium^®^ MethylationEPIC BeadChip (Illumina Inc., San Diego, CA, USA)
				CIMP+	hyper	better	CIR_3yr_ = 15%		

#, Survival and relapse rates—only statistically significant findings from each study are reported in the table; ##, survival and relapse rates estimated from a supplementary figure of the reviewed publication; *, 24 patients studied by Me-DIP; **, 168 patients (validation cohort) studied by MS-MLPA; MRD, minimal residual disease; n.a., not available information on treatment protocol—patients treated within Tokyo Children’s Cancer Study Group (TCCSG) and the Japan Association of Childhood Leukemia Study (JACLS); CIMP, CpG Island Methylator Phenotype; COSMe, CpG island and Open Sea Methylation; hypo, hypomethylated phenotype; hyper, hypermethylated phenotype; OS, overall survival; DFS, disease-free survival; EFS, event-free survival; CIR, cumulative incidence of relapse; MS-PCR, methylation-specific polymerase chain reaction; Me-DIP, methylation-dependent immunoprecipitation; MS-MLPA; methylation-specific multiplex ligation-dependent probe amplification.
